# Laser Application in Life Sciences

**DOI:** 10.3390/ijms24108526

**Published:** 2023-05-10

**Authors:** Herbert Schneckenburger

**Affiliations:** Institute of Applied Research, Aalen University, Beethovenstr. 1, 73430 Aalen, Germany; herbert.schneckenburger@hs-aalen.de

## 1. Introduction

Since their invention by Theodore Maiman in 1960, lasers represent a class of light sources based on the stimulated emission of radiation in the visible, ultraviolet or infrared spectral range. Due to this principle, laser light possesses specific and unique properties, e.g., high coherence, low spectral bandwidth, excellent focusing properties and often a concentration in short pulses, which make lasers indispensable tools in the life sciences. Lasers emit light of one or several discrete wavelengths, or may be tuned over a large spectral range. They are operated either in a continuous wave (cw) or in a pulsed mode, with pulse durations ranging from the millisecond to the femtosecond range. The pulse duration primarily determines the interaction of lasers with cells or tissues. If an energy density of a few J/cm² is applied continuously, laser light is often well tolerable and non-destructive to living cells or tissues. It is absorbed or scattered, and can be used for diagnostic purposes, e.g., fluorescence or Raman spectroscopy. However, in some cases, cw light of several J/cm² may induce photochemical reactions of light-sensitive molecules and can be used for photosensitization and photodynamic therapy (PDT) of cancer and other diseases [1,2]. Laser pulses of some J/cm² applied in the microsecond–millisecond range primarily induce thermal reactions, which are favorable for coagulation (e.g., of the retina [3]) or evaporation (cutting) of tissue in various kinds of operation. In contrast, laser pulses of similar energy density, applied in the nanosecond–femtosecond time range, induce less thermal reactions, but due to opto-mechanical interactions, can be applied for tissue ablation, microdissection [4], hole drilling or laser-assisted optoporation [5,6]. Papers of the Special Issue “Laser Application in Life Sciences 2018” encompass research topics relating most kinds of laser applications.

## 2. Overview of Submitted Articles

The fields of application within this Special Issue range from molecular and cellular diagnostics [7,8] low-level laser therapy (photobiomodulation) [9], through to the treatment of dentin with high-energy laser pulses [10]. The potential of cell diagnosis is improved by measuring molecular distances in the nanometer range using super-resolution microscopy [11]. Laser-assisted micromanipulation and microdissection is described in view of isolation and analysis of cellular protrusions, and an understanding of their possible role in the life sciences [12]. While photodynamic therapy (PDT) is a well-established method for therapy of human cancer, this technique had been described in two papers about application to a fungal pathogen (*Candida albicans*) [13,14].

The article by Kitamura et al. [7] provides a review on spectroscopic techniques used for the study of protein aggregates and their specific foldings, which are characteristic of neurodegenerative diseases, e.g., Alzheimer’s disease, Parkinson’s disease or Huntington’s disease. Techniques are often based on the fluctuations of fluorescence, e.g., in fluorescence correlation spectroscopy (FCS) or image correlation spectroscopy (ICS), super-resolution optical fluctuation imaging (SOFI) or transient state (TRAST) monitoring spectroscopy. However, many relevant molecules (e.g., β-amyloid peptides) do not fluoresce in the visible part of the spectrum and are, therefore, labelled with organic dyes or green fluorescent proteins (GFPs). Since the methods are based on molecular diffusion, they support the analysis of the aggregation process. SOFI has been established as a single-molecule localization method with a resolution below the Abbe criterion, and competes with established methods such as stochastic optical reconstruction microscopy (STORM), photo-activated localization microscopy (PALM) or stimulated emission depletion (STED) microscopy. TRAST is used to measure transient triplet or radical states, as well as light-induced isomerization or conformational molecular changes after excitation with various laser pulse lengths.

The paper by Leben et al. [8] additionally describes a potential spectroscopic method: fluorescence lifetime imaging microscopy (FLIM). This method is useful for distinguishing fluorescent molecules of different conformation or different interaction from their molecular or cellular environment. Here, the co-enzymes nicotinamide adenine dinucleotide and nicotinamide adenine dinucleotide phosphate (NAD(P)H), which are not further distinguished due to their almost identical fluorescence spectra and lifetimes, are used as metabolic indicators of various enzymatic reactions occurring, e.g., upon oxidative phosphorylation or during phagocytosis. Since a “free” form of NAD(P)H with a fluorescence lifetime of 0.4–0.5 ns and a protein/enzyme bound form with a lifetime of 1.5–2.5 ns coexist, these forms can be distinguished through fluorescence decay analysis and described by a “phasor plot”. Data are analyzed to evaluate the mechanisms of cell death of human neutrophil granulocytes upon the phagocytosis of *Staphylococcus aureus* beads, and may be further used in a broader field of immunology to monitor the basic mechanisms of cell and tissue functions. In the present studies, two-photon excitation in a laser-scanning microscope confines the measured volume and suppresses the fluorescent background.

Super-resolution localization microscopy of single molecules and molecular distances in cells, and particularly the cell nucleus, are the subject of the article by Hausmann et al. [11]. The authors use various kinds of staining-specific receptors or antibodies, and measure molecular distances in the nanometer range. Of particular interest are responses to cancer treatment, e.g., by chemotherapeutic drugs or ionizing radiation. Here, the authors prove the expression of specific molecules or genes, as well as the re-organization of the chromatin structure. In addition to advanced cancer diagnosis, the studies aim to better understand the cellular characteristics in tumor genesis and the novel treatment procedures for personalized medicine.

Tani et al. [9] describe low-level laser therapy at energy densities below 10 J/cm^2^, where neither photochemical nor photothermal reactions would be expected. Nevertheless, they find stimulating effects on human osteoblasts and mesenchymal stromal cells upon cw illumination with red (635 ± 5 nm), near-infrared (808 ± 10 nm) or violet-blue light (405 ± 5 nm) from a diode laser (635 nm, 808 nm) or an LED (405 nm). These effects concern cell viability, proliferation, adhesion and differentiation, and are summarized as “photobiomodulation” (PBM). The experiments suggest that a PBM with 635 nm may be a potential option for promoting or improving bone regeneration; however, more precise light dose studies are to be performed, since at higher light doses, the biological stimulation may disappear or even be replaced by inhibition.

In view of an efficient caries prevention, the article by Pereira et al. [10] describes the treatment of dentin with fluoride compounds, high-energy laser pulses, as well as a combination of both. Irradiation occurs with pulses of a Nd:YAG laser at a wavelength of 1064 nm, an energy density of 85 J/cm^2^, a pulse duration of 100 µs and a frequency of 10 Hz. Dentin surfaces are evaluated with respect to their composition by attenuated total reflectance Fourier transform infrared spectroscopy (ATR-FTIR), their crystalline structure via X-ray analysis and their morphology using electron microscopy, as well as optical coherence tomography (OCT). It was concluded that Nd:YAG laser irradiation promotes the melting and recrystallization of the dentin surface, and prevents dentin erosion, as well as the abrasion process.

While the irradiation of dentin may occur on larger surfaces (300 µm spot size in [10]), extreme focusing is required in laser-assisted micromanipulation and microdissection. In [12], a UV laser is focused to a 0.3–0.5 µm diameter spot, which is moved with a speed of 10–35 µm for cutting cellular protrusions. Laser capture microdissection (LCM) does not only cut cells and organelles, but combines them with fixation, protein extraction and identification via mass spectrometry. Thus, the authors can isolate different subtypes of protrusions with distinct proteomes, which may help understand the possible role of these unique structures in health and disease. Unfortunately, the paper is lacking data on laser wavelength, pulse duration and repetition rate, which would further elucidate the mechanisms involved.

Finally, two articles [13,14] are dedicated to photodynamic inactivation (PDI) or photodynamic therapy (PDT) of the fungal pathogen *Candida albicans*, which often grows as a biofilm on mucosal surfaces and may cause tissue infections in the skin, mucosal oral cavity, gastrointestinal tract, vagina and even the bloodstream of humans. Biofilms of *C. albicans* are incubated with a photosensitizing agent and exposed to moderate light doses (50 J/cm^2^ at 630 nm [14] or 9 J/cm^2^ at 430 nm [13]). The authors show that a combination of PDT (or PDI) and application of antifungal agents reduces cell viability considerably. Therefore, it appears promising to use this combination even clinically for treatment against *C. albicans* infections.

## 3. Concluding Remarks

This Editorial reflects state-of-the-art of laser applications in life sciences in 2018. The papers are partially concentrated on microscopy [15], and it appears that in this field, much of the progress was reported in the last few years concerning 3D imaging (see e.g., [16]), super-resolution microscopy (see [17] or FLIM (see [18]). However, in addition to all new scientific results, the clinical application of laser-based methods will attain an essential role.
